# Roles of Carotenoids in Invertebrate Immunology

**DOI:** 10.3389/fimmu.2019.03041

**Published:** 2020-01-17

**Authors:** Karsoon Tan, Hongkuan Zhang, Leong-Seng Lim, Hongyu Ma, Shengkang Li, Huaiping Zheng

**Affiliations:** ^1^Key Laboratory of Marine Biotechnology of Guangdong Province, Institute of Marine Sciences, Shantou University, Shantou, China; ^2^Mariculture Research Center for Subtropical Shellfish & Algae of Guangdong Province, Shantou, China; ^3^STU-UMT Joint Shellfish Research Laboratory, Shantou University, Shantou, China; ^4^Borneo Marine Research Institute, University Malaysia Sabah, Kota Kinabalu, Malaysia

**Keywords:** carotenoids, invertebrate, immunity, antioxidant, immunostimulant, review

## Abstract

Carotenoids are biologically active pigments that are well-known to enhance the defense and immunity of the vertebrate system. However, in invertebrates, the role of carotenoids in immunity is not clear. Therefore, this study aims to review the scientific evidence for the role of carotenoids in invertebrate immunization. From the analysis of published literatures and recent studies from our laboratory, it is obvious that carotenoids are involved in invertebrate immunity in two ways. On the one hand, carotenoids can act as antioxidant enzymes to remove singlet oxygen, superoxide anion radicals, and hydroxyl radicals, thereby reducing SOD activity and reducing the cost of immunity. In some organisms, carotenoids have been shown to promote SOD activity by up-regulating the expression of the *ZnCuSOD* gene. Carotenoids, on the other hand, play a role in the expression and regulation of many genes involved in invertebrate immunity, including thioredoxins (TRX), peptidoglycan recognition receptor proteins (PGRPs), ferritins, prophenoloxidase (ProPO), vitellogenin (Vg), toll-like receptor (TLRs), heat shock proteins (HSPs), and CuZnSOD gene. The information in this review is very useful for updating our understanding of the progress of carotenoid research in invertebrate immunology and to help identify topics for future topics.

## Introduction

Vertebrates possess both innate and adaptive immune systems, of which the adaptive immune system has many specialized cells and molecules that interact in a particular way (1). Unlike vertebrates, the only line of defense for invertebrates is innate immune system (2). The response of innate immunity to infection or injury is by releasing highly reactive oxygen and nitrogen species (ROS and RNS) at the focal point, which leads to immune cost and host tissues destruction (immunopathology) (3, 4). The cost of immunity is the operating cost of the immune response, which reduces the availability of resources for other physiological functions (5, 6). Being lack of specific immunity, both enzymes and non-enzymatic antioxidants play a crucial role in immunity of invertebrates (7). The main enzymes in the innate immune system include catalase (CAT), superoxide dismutase (SOD), glutaredoxins, thioredoxins (TRX), peroxiredoxins (PRXs), and GSH-Px (8). Major non-enzymatic antioxidants, including carotenoids, polyunsaturated fatty acids (PUFA), uric acid, vitamins (vitamins C and E) and GSH, and a tripeptide (L-g-glutamyl-L-cysteinyl-L-glycine), comprise a thiol (sulfhydryl) group (8, 9).

**Figure 1:**
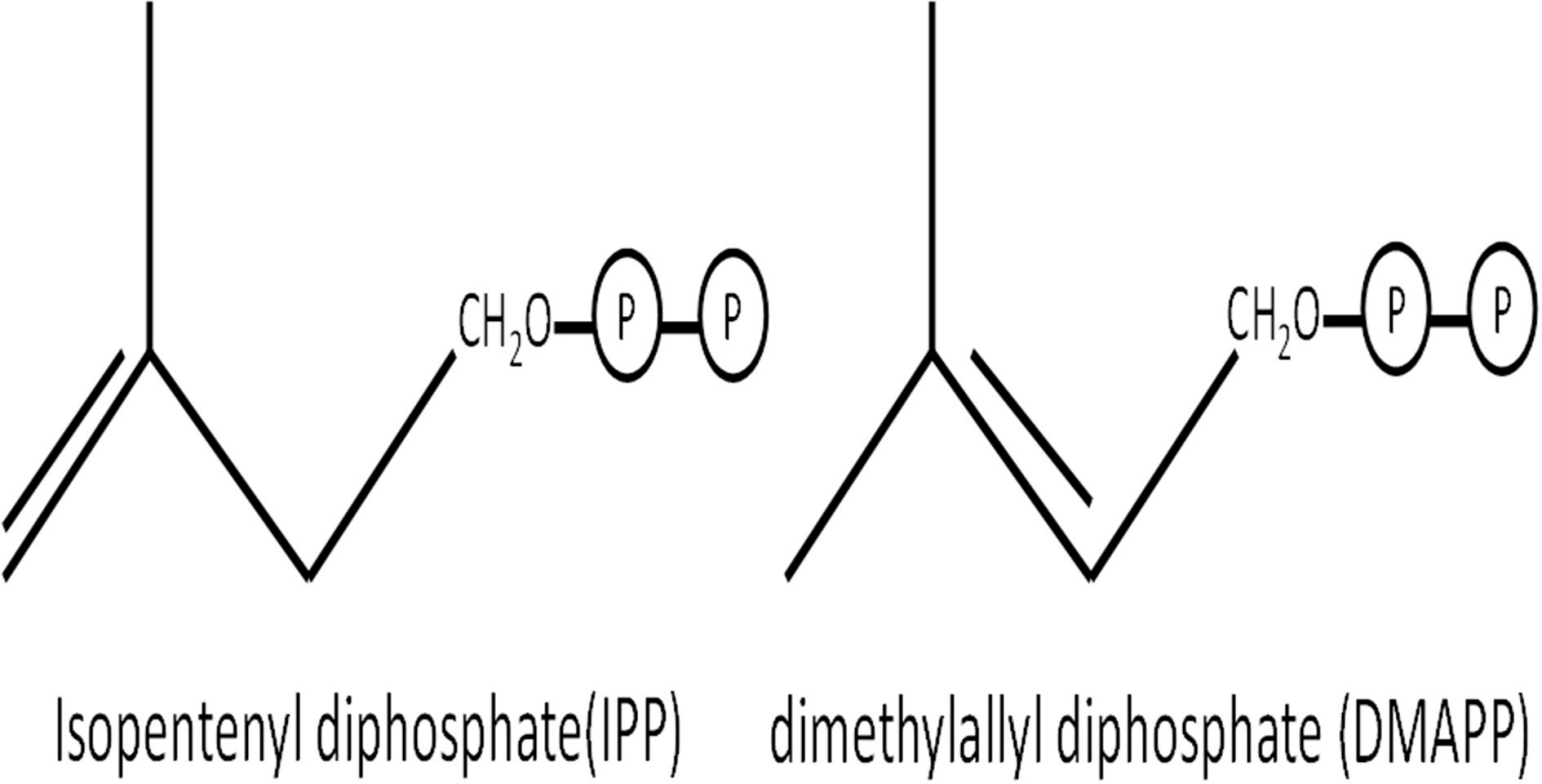
Basic blocks of carotenoids.

Carotenoids, also known as tetraterpenoids, are responsible for colorless, red, orange, and yellow pigments in plants, insects, crustaceans, fish, and birds (10–12). Carotenoids are produced by plants, algae, and certain microbes (10), with basic structural units of isopentenyl (IPP) and dimethylallyl diphosphate (DMAPP) (Figure 1). Carotenoids are biologically active pigments that have beneficial effects on the body conditions (13), which increase the efficiency of immune responses and stimulate innate immunity components (14, 15). Furthermore, as part of the integrated antioxidant system (16, 17), high carotenoid content in an organism enhances immunity without increasing the associated costs of immunity by aiding endogenous enzymes (e.g., catalase, superoxide dismutase) and detoxifying free radicals produced during immune activity (18–21). In general, animals cannot biosynthesize carotenoids *de novo*, so they obtain carotenoids either directly from food or partially modified through metabolic reactions (22).

On the other hand, Royet et al. (23) revealed a series of pattern recognition receptors (PRR), especially peptidoglycan recognition proteins (PGRPs), which are highly conserved in evolution and can help the innate immune system recognizing pathogens through their unique cell wall component, peptidoglycan (PGN). The ability of carotenoids in regulating the transcription of various PRR genes has been extensively studied in vertebrates, particularly in humans (24, 25). Moreover, the immune response of invertebrates to infectious diseases has received considerable attention because many of them are important species in fisheries and aquaculture, while others are critical to the structure and function of ecosystems (26–39).

To date, Maoka (22), Liaaen-Jensen (40, 41), Matsuno (42, 43), and Matsuno and Hirao (44) have reviewed carotenoid species in invertebrates. All of these published reviews have focused on the emergence of specific carotenoid groups in invertebrates, but the links between carotenoids and invertebrate immunity have received relatively little attention. Recently, there have been more and more reports on the roles of carotenoids in invertebrate immunity in time-series observations and laboratory experiments (26, 35–38, 45–51), but the information is not well-collated. Therefore, in this review, we summarized the currently available data on the effects of carotenoids on invertebrate immunity. In particular, we reviewed the role of carotenoids in antioxidant system, immune system, and unfavorable environmental tolerance of invertebrates. To the best of our knowledge, this paper represents the first review reviewing the role of carotenoids in invertebrate immunity. This information is very useful for updating our understanding of the progress of the carotenoid research in invertebrate immunology and for identifying future research topics.

## Invertebrate Immune System

Invertebrates account for 97% of animal diversity and can actually be found in any environment. In general, a range of cellular and humoral defenses are involved in protecting invertebrates from pathogens that manage to penetrate their exoskeleton/cuticle or alimentary canal to internal tissues. Cells associated with innate immunity include hemocytes (circulating and sessile blood cells) and various other cell types, including fat body, coelomocytes, hepatopancreas, and gills in insects, earthworm, molluscs, and crustaceans, respectively (52–55). If the pathogens manage to penetrate its external barrier, the blood cells present in the body cavity can destroy the tiny invaders such as bacteria, fungi, protozoans, and viruses by phagocytosis, and encapsulate the multicellular parasites by encapsulation, thereby isolating invaders from the host (54–56).

In the humoral defense of invertebrates, invaders such as bacteria and fungi are eliminated by antimicrobial peptides (AMPs). In insects (*Drosophila*) and shrimps, several distinct AMP forms are synthesized by the fat body and hemocytes, respectively (57). The prophenoloxidase (proPO) enzyme cascade based on phenoloxidase (PO) enzyme activity is one of the effective effectors of humoral responses. After a stepwise proteolytic activation, the PO enzyme is responsible for the production of melanin, as well as the release of quinone-derived metabolites and reactive oxygen species. These chemicals are not only destructive to pathogens, but also have cytotoxic oxidative effects on the basic host cell components (58, 59). Matova and Anderson (60) reassessed the importance of cellular (phagocytosis) and humoral (AMP) defense in *Drosophila*. The results indicated that the double mutants of *Drosophila* larvae contain negligible circulating hemocytes, but high levels of AMPs did not survive from opportunistic bacterial or fungal infection (60).

Invertebrates are a very heterogeneous group of animals (about 1.3 million species). This wide distribution indicates that innate immune defense mechanisms of invertebrates enable them to adapt and survive in diverse environments. In fact, in the same host, different bacterial strains or species may trigger different immune effectors, leading to different immune responses (61). It is worth noting that recent studies in sea urchin have shown that titanium dioxide nanoparticles can temporarily suppress the inflammatory-related gene transcription and boost metabolic activity of antioxidants (62–64). The innate immune memory in invertebrates such as bivalves (65), gastropods (66), insect (67), and crustaceans (68) is established by re-programming of innate immune functions after being induced by a stimulus, which will result either in decreased reactivity (tolerance) or increased responsiveness (potentiation) to a subsequent challenge. In both cases, the main purpose of establishing innate immune memory is to better defend and regulate its functional immune phenotype in response to subsequent stimuli.

## Carotenoids

Carotenoids have received considerable attention for their beneficial effects on human health and their wide range of biotechnological applications (11, 69). Carotenoids have been reported to enhance the immune system (70), repair DNA damage (51), and prevent *in vitro* auto-oxidative damage of human lymphocytes (71), thereby reducing the risk of various diseases (72, 73).

Some invertebrates, including insects, polyplacophora, echinoderms, gastropods, bivalves, and cephalopods, are rich in carotenoids (35, 48, 49, 74). The accumulation of carotenoids in invertebrates is tissue specific, and the highest total carotenoids content (TCC) is usually observed in the gonads, as carotenoids are essential for invertebrate reproduction (74). Zheng et al. (74) have shown that the accumulation of TCC in invertebrates, particularly bivalve, noble scallops *Chlamys nobilis*, is affected by genetic factors, which are controlled by genes associated with carotenoid absorption, such as *SRB-like-3* (75) and *StAR-like-3* (76).

Chemically, the polyene backbone consists of a series of conjugated C=C bonds. This particular feature is responsible for the main biological functions of carotenoids associated with antioxidant properties, where dietary carotenoids provide a degree of antioxidant protection for cells, tissues, and other structures (24, 51, 77), thereby reducing self-harming caused by cytotoxic chemicals released by immunological activity (78, 79). Moreover, carotenoids can enhance the defense capability and immune competence of various animal systems by up-regulating the expression levels of immune-related genes (35–38, 80).

## Roles of Carotenoids in Invertebrate Antioxidant System

**Figure 2:**
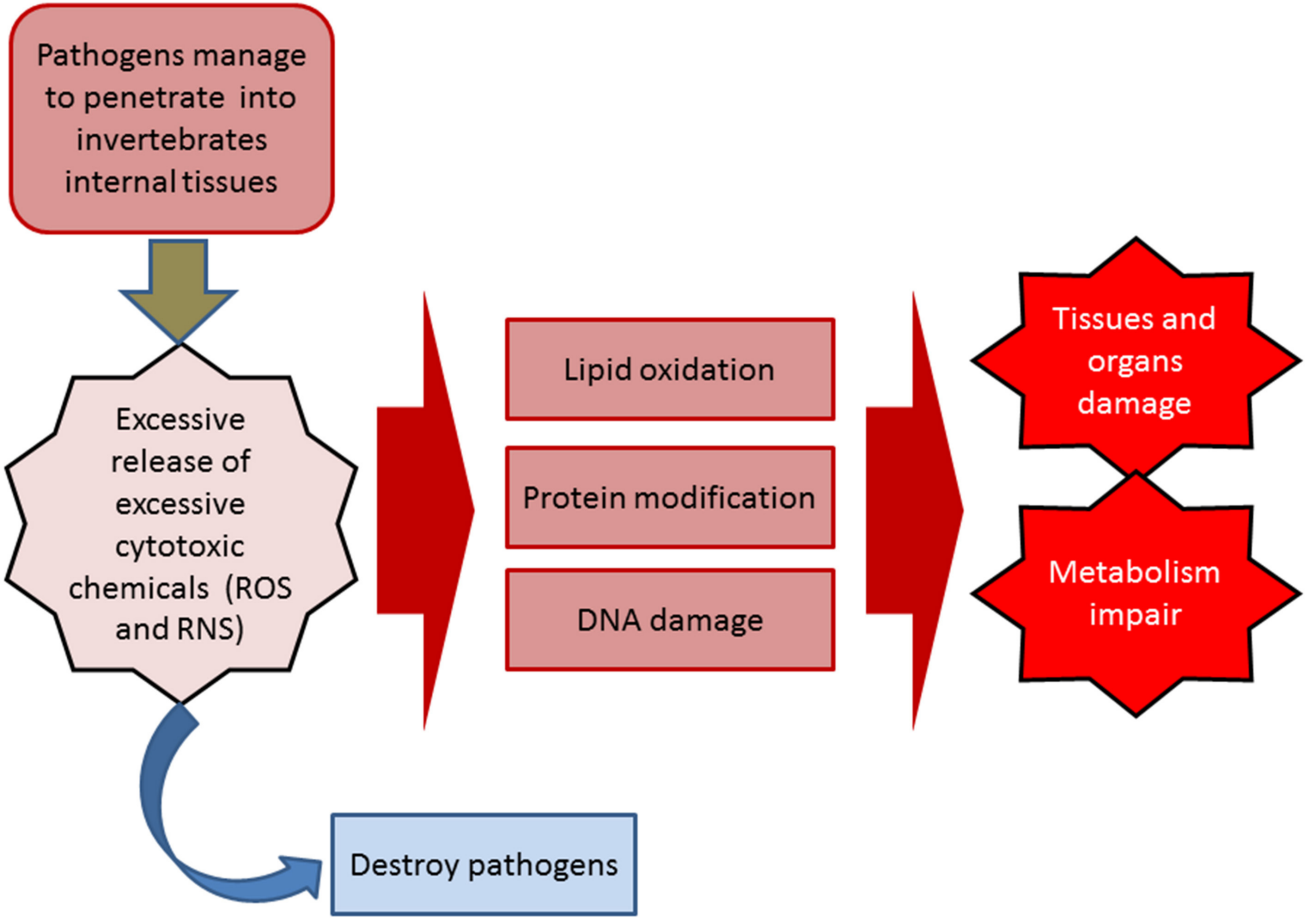
Inflammatory response in innate immunity.

During the inflammatory response, the release of excessive cytotoxic chemicals [highly reactive oxygen species (ROS) and nitrogen species (RNS)] not only destroys pathogens and parasites, but also damages the tissues and organs of the host itself (immunopathology) (3). These biochemical and physiological damages may eventually lead to disease by impairing metabolism, causing oxidative damage to lipids, proteins, and nucleic acids (4, 81–83) (Figure 2). In addition, if the damaged tissues are not fully recovered and that homeostasis is not restored, it will further develop into a chronic condition, such as an increase in rates of morbidity and mortality in the elderly (84–86). Antioxidant defense systems in invertebrates play a vital role in controlling the amount of circulating cytotoxic ROS and RNS. This system comprises three key antioxidant enzymes, including superoxide dismutase (SOD), catalase (CAT), and glutathione peroxidase (GP), which in turn participate in the detoxification of superoxide radicals: SOD converts superoxide into hydrogen peroxide, which is then detoxified into water and oxygen by CAT and GP (17, 87). It has been reported that the structure and function of SOD are well-conserved in diverse organisms including marine invertebrates (88).

**Figure 3:**
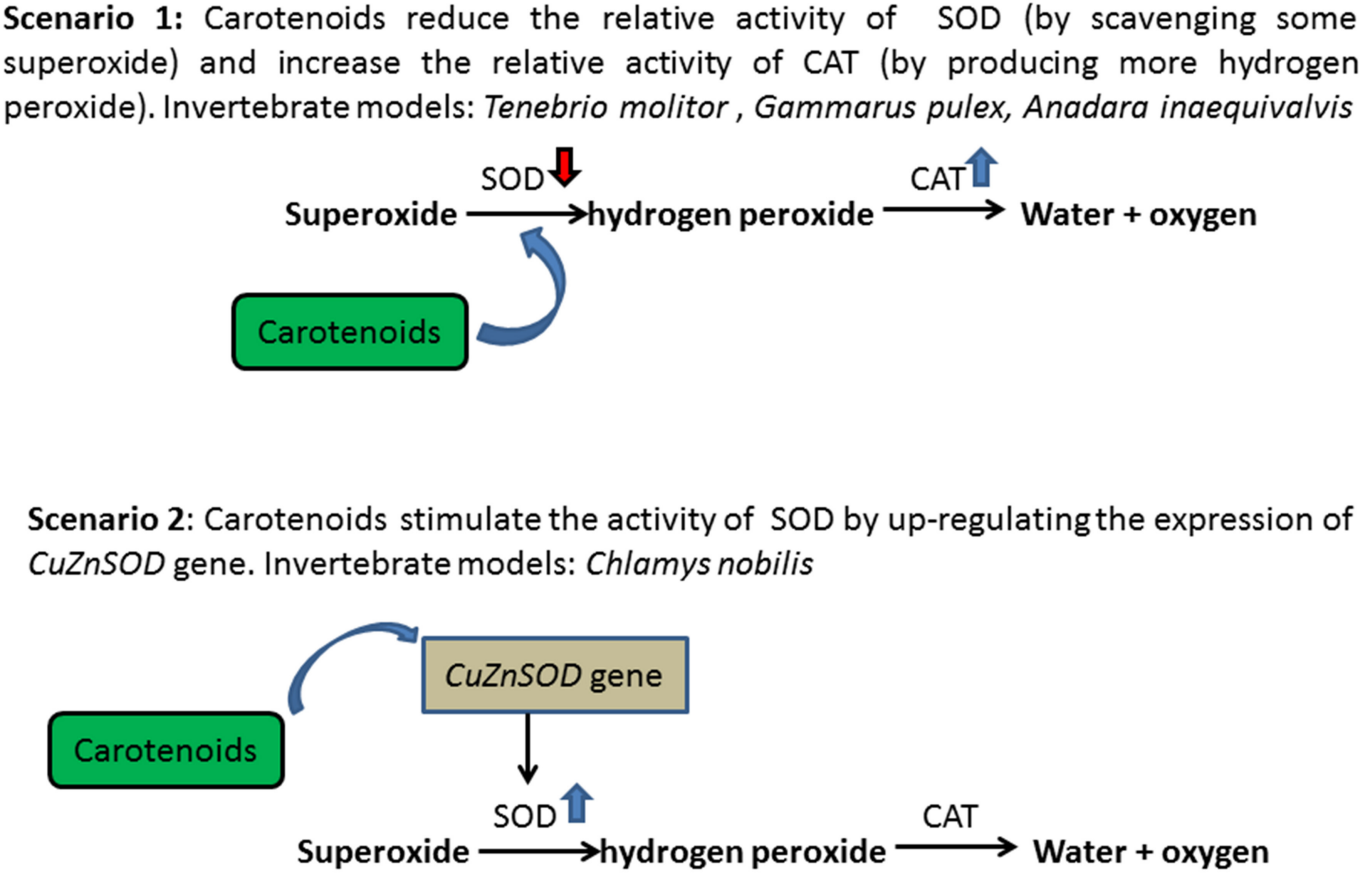
Roles of carotenoids in invertebrate antioxidant system.

In invertebrates, carotenoids are involved in the antioxidant defense system in two ways (Figure 3). On the one hand, carotenoids may reduce the relative activity of antioxidant enzymes by taking over their actions, thereby reducing the cost of immunity (20, 21). Carotenoids are effective in scavenging singlet oxygen (^1^O_2_) (89), superoxide anion radicals (SOAR), and hydroxyl radicals (OH**•**) (90). Tsushima et al. (91) reported that the β-carotene supplementation in cultured sea urchin significantly increased carotenoids levels in the gonads, thereby enhancing the immunological activity of sea urchins and was associated with higher reproduction and survival of sea urchin larvae (92). Invertebrates that store large amounts of carotenoids in tissues have a higher competitive relationship with the cellular enzymatic antioxidant (AO) complexes for the corresponding substrates. Dhinaut et al. (48) used the mealworm beetle *Tenebrio molitor* as a biological model of invertebrates, demonstrating that lifetime food supplementation with carotenoids (particularly astaxanthin) can prevent immunopathology through immunosuppression. Moreover, Babin et al. (49) revealed that the presence of considerable concentration of carotenoids in amphipod crustacean *Gammarus pulex* reduced SOD activity, in which carotenoids take over the role of SOD, first acting on the detoxification chain of superoxide radicals and producing hydrogen peroxide. Then, a high concentration of hydrogen peroxide promotes CAT activity, acting in second in the detoxification chain. A similar observation has been reported in the blood cockles, *Anadara inaequivalvis*, in which 1.7 to 2.9 times lower SOD activity and an elevated content of reduced glutathione were recorded in tissues with high carotenoid content (50). In addition, carotenoids can reduce the susceptibility of single-stranded nucleic acid breaks in cell lines to oxidative damage (100 μmol H_2_O_2_/L, 5 min, 4°C), either by scavenging DNA-damage free radicals or regulating DNA repair mechanisms (51).

On the other hand, besides a free radical scavenger, carotenoids have also been shown to stimulate the activity of antioxidant enzymes in invertebrates (38), thereby enhancing the detoxification efficacy of immunological activity. It has been reported that in noble scallops, *C. nobilis, CuZnSOD* gene is up-regulated by carotenoid to enhance detoxification (38). However, due to the continuous contact of gills with water, the up-regulation of *CuZnSOD* transcript is tissue-specific, with the highest expression levels in gills (93). In addition, it is evident from clinical studies that a high-fat diet with high carotenoid content is associated with an increase in CuZnSOD, thereby increasing the expression of CuZnSOD and protecting tissue cells from oxidative damage (94). In both cases, carotenoids can improve the detoxification of free radicals and reduce the costs associated with oxidative stress.

## Role of Carotenoids as Immunostimulant in Invertebrates

Carotenoids enhance the defense and immunity of various animal systems (18, 95). For instance, dietary trials in amphipod crustacean *G. pulex* demonstrated that supplementation of astaxanthin resulted in broad stimulation of innate immunity to gammarid and increase resistance to microbial infection (18). Similarly, Kumar et al. (95) revealed that dietary supplementation of astaxanthin was associated with an increase in phenoloxidase activity and total hemocyte count in the giant freshwater prawn *Macrobrachium rosenbergii*. In the same species, the injection of astaxanthin also increased the total hemocyte count and survival rate under the challenge of the pathogenic bacterium *Lactococcus garvieae* (96). The higher immunity of invertebrates containing significant amounts of carotenoids can be partly explained by the fact that carotenoids regulate gene expression of several immune-related genes, in particular thioredoxin-like protein (TRX) gene (35), peptidoglycan recognition receptor proteins (PGRPs) gene (Unpublished data), ferritin genes (47), prophenoloxidase (ProPO) gene (18, 39), vitellogenin (Vg) gene (36), toll-like receptor (TLRs) gene (37), Heat shock proteins (HSP70 and HSP 90) (26, 45, 97), and CuZnSOD gene (38) (Table 1).

**Table 1:** Gene expression regulations by carotenoids under different stressors.

**Gene**	**Up-regulated gene expression (times)**	**Stressors**	**References**
Thioredoxins (TRX)	1.18–1.76	*Vibrio parahaemolyticus*	(35)
PGRP gene	1.60–1.80	*V. parahaemolyticus* and polyinosinic polycytidylic acid (Poly I: C)	Unpublished data
Ferritins	1.20–1.90	*V. parahaemolyticus*	(47)
Prophenoloxidase (ProPO)	1.07–1.21	Bacterial infection	(18, 39)
Vitellogenin (Vg)	1.60–2.78	NiL	(36)
Toll-like Receptor (TLRs)	1.10–4.00	*V. parahemolyticus* and lipopolysaccharide and Poly I:C	(37)
Heat shock proteins (HSPs)	1.20–2.00	Heat shock challenge at 32°C	(26, 45)
CuZnSOD gene	1.20–2.20	lower temperature stress	(38)

Thioredoxins (TRXs) contain a dithiol/disulfide active site (CGPC) and are the major cellular protein disulfide reductase in the thioredoxin system (98). TRXs have been shown to be involved in the immunity of marine invertebrates, including *Apostichopus japonicas* (99), *Ruditapes philippinarum* (100), *Litopenaeus vannamei* (101), and *Haliotis discus discus* (102). Up-regulation of TRX gene expression in bivalves has been demonstrated under bacteria stress (35, 103). Recent studies on the effects of *Vibrio parahaemolyticus* challenge on two polymorphic scallops with different total carotenoid content (golden and brown scallops; the total carotenoid content of golden scallops is significantly higher than that of brown scallops) reveal that the expression level of *TRX* is up-regulated in the golden scallops by 1.18 to 1.76 times relative to the brown scallops, indicating that carotenoids up-regulated the expression of *TRX* gene under bacterial challenge (35).

Vg is a precursor of vitellin (Vn) in egg yolk, a major source of energy in embryonic development (104). Vg is expressed in females of nearly all oviparous species, including amphibians, fish, birds, reptiles, most invertebrates, and monotremes (105, 106). Moreover, Vg is a non-polar molecule carrier that transfers lipids and carotenoids to oocytes (107). The role of Vg in host immune defense against bacteria and viruses has been extensively studied in many oviparous animals, including marine bivalve *Patinopecten yessoensis*, in which Vg has antibacterial activity (108). Zhang et al. (36) demonstrate that the Vg transcription level in the ovary of noble scallop *C. nobilis* is directly proportional to the total carotenoid content, indicating that Vg expression is up-regulated by carotenoids.

PGRPs are a group of pattern recognition receptors (PRRs) that are conserved from invertebrates to vertebrates. However, PGRPs function differently in innate immunity of invertebrates and vertebrates. In most invertebrates, PGRPs not only participate in multiple host defense processes, including hydrolysis of PGN and cell phagocytosis, but also play an important role in bacterial pathogen sensing (109, 110). For example, in bay scallops, PGRP is involved in the scallop immune response against gram-positive bacterial infections, primarily induced by bacterial PGN (109). In *Solen grandis*, both PGRP-S1 and PGRP-S2 are induced by pathogen-associated molecular patterns (PAMPs) and PGN (110), whereas in *C. nobilis*, the *CnPGRP* gene can be induced by *V. parahaemolyticus*, LPS, and Poly I:C. Interestingly, the presence of high total carotenoids content was associated with up-regulation of *CnPGRP* expression, particularly in the gills (1.8 times) and hepatopancreas (1.6 times) of *C. nobilis* under immunostimulant stress (unpublished data).

Ferritins are important iron-chelating proteins that play crucial roles in the iron-withholding defense system (111). These proteins are ubiquitous in a variety of organisms, including fungi, bacteria, invertebrates, plants, and vertebrates, and show several conserved features (112). High expression of cytosolic and secreted ferritin in invertebrates has been demonstrated to improve innate immune defense in invertebrates (113). Under bacterial challenge, the expression level of *ferritins* gene (up-regulated by 1.20–1.90 times) was positively correlated with total carotenoids content in *C. nobilis*, indicating that carotenoids up-regulated the expression of *ferritins* gene under bacterial stress (47).

Pattern recognition receptors (PRRs) are a group of recognition proteins of the innate immune system associated with the detection of pathogen-associated molecular patterns (PAMPs) (114, 115). One type of PRR, TLRs, is an important transmembrane protein that connects innate immunity and adaptive immunity (116). TLRs detect microorganisms based on conserved PAMPs such as lipoproteins, PGNs, lipopolysaccharides (LPS), double-strand viral RNA, unmethylated bacterial CpG DNA, and many more (117). In non-mammalian vertebrates to invertebrates, many species have also been shown to have numerous TLRs (118). Invertebrate TLRs are mainly studied in bivalves such as *Chlamys farreri* (119), *Mytilus galloprovincialis* (120), *Crassostrea gigas* (121), *Mya arenaria* (122), the cephalopod *Sepia officinalis* (123), and *Euprymna scolopes* (124). Laboratory challenge tests on polymorphic scallops with *V. parahemolyticus* and LPS and Poly I:C showed that under the influence of carotenoids, *CnTLR-1* transcripts were significantly up-regulated in the hemolymph (37).

Prophenoloxidase (ProPO) activation system plays an important role in initiating melanin synthesis by detecting microbial cell surface molecules such as PGNs or LPS in bacteria and β-1,3-glucans in fungi (125–127). Cornet et al. (39) found that both PO and total activity were positively correlated with carotenoid concentrations in the hemolymph of *G. pulex* population, highlighting the potential importance of dietary carotenoids in the evolution of investments in immune defense and their short-term up-regulation in *G. pulex*. Consistent with this up-regulation, dietary supplementation with carotenoids is associated with increased resistance to bacterial infections, further supporting the idea of stimulating the effects of carotenoids on immunity. Babin et al. (18) propose that this immunostimulation either requires the carotenoids in the hemolymph to reach a threshold level or that it needs to increase the level of carotenoids for a sufficiently long time. Unexpectedly, despite the enhanced immunological activity, gammarids fed on carotenoids did not suffer from additional immunity cost compared to control gammarids (18). Raising PO activity is likely beneficial in fighting pathogen attacks, but it is also known to be costly through autoreactivity (4). Therefore, this observation might indicate that dietary carotenoids help to reduce this cost, enabling individuals to raise their immune activity and resist infections more effectively.

## Role of Carotenoids in Invertebrate Environmental Tolerance

Immunity is strongly influenced by environmental conditions. Altered environmental conditions can directly affect immunity by changing the concentration and efficiency of cytokine receptors, cytokines, and cells of the immune response, or indirectly affecting immunity by inducing general stress responses (128). Carotenoids have increased the resistance of invertebrates to environmental stress factors such as low salinity (46) and heat stress (26, 38, 45, 97). Under low salinity stress, the SOD content and expression level of scallop serine protease inhibitor (SPI) gene were positively correlated with total carotenoids, indicating that carotenoids enhanced the resistance of *C. nobilis* to low salinity (46).

Heat shock proteins (HSPs) are composed of a group of highly conserved proteins that are widely found in prokaryotes and eukaryotes. They increase the resistance of organisms to stressors and maintain cellular homeostasis (129). HSP expression is rapidly up-regulated when organisms are exposed to hypoxia, high temperatures, heavy metals, pathogen invasions, starvation, or trauma (130, 131). The HSP90 synthesis has been shown to induce by external stress in various mollusks, such as *C. farreri* (132), *Argopecten irradians* (133), *C. gigas* (134), *Haliotis discus hannai* (135), *R. philippinarum* (136), and *Hyriopsis cumingii* (129). Recent studies have demonstrated that in a heat shock challenge at 32°C for 36 h, *HSP90* expression levels are higher (1.2 to 2.0 times) in *C. nobilis* with higher total carotenoids content (60 to 120 μg/g) compared with common brown noble scallops with lower TCC (40 to 52 μg/g) (97). In the same species, carotenoids can also up-regulate the expression of *HSP70* by 2.0 to 12.0 times after 36 h of acute cold stress challenge in 8°C (26) and acute heat stress challenge in 32°C (45).

Carotenoids also have shown to up-regulate the expression of *CuZnSOD* gene in mollusc under low temperature stress, and there was a strong positive correlation between total carotenoid content and the expression level of *CuZnSOD* gene in *C. nobilis* (38). Moreover, carotenoids can also enhance defense by participating in maintenance of membranes in a fluid state during low-temperature stress (137).

## Conclusion

Evidence from analytical, biochemical, and molecular studies demonstrated that carotenoids play an important role in invertebrate immunity. Carotenoids not only actively scavenge singlet oxygen, superoxide anion radicals, and hydroxyl radicals, thereby reducing the cost of immunity, but also regulate the expression of immune-related genes. In this context, there are still some issues that need to be resolved. The exact mechanism by which carotenoids may reduce the cost of self-harm in immune responses remains to be tested. Moreover, the exact role of carotenoids in more invertebrate species remains to be studied.

## Author Contributions

KT performed the data analysis and drafted the manuscript. HZhe, HZha, L-SL, HM, and SL revised the draft.

### Conflict of Interest

The authors declare that the research was conducted in the absence of any commercial or financial relationships that could be construed as a potential conflict of interest.
